# Hypoglycemic Effect of *Opuntia ficus-indica* var. *saboten* Is Due to Enhanced Peripheral Glucose Uptake through Activation of AMPK/p38 MAPK Pathway

**DOI:** 10.3390/nu8120800

**Published:** 2016-12-09

**Authors:** Kang-Hyun Leem, Myung-Gyou Kim, Young-Tae Hahm, Hye Kyung Kim

**Affiliations:** 1 College of Korean Medicine, Semyung University, Chungbuk 27136, Korea; heavenok@dreamwiz.com (K.-H.L.); ehdfkd@hanmail.net (M.-G.K.); 2 Department of Biotechnology, Chung-Ang University, Gyeonggi 17546, Korea; ythahm@cau.ac.kr; 3 Department of Food & Biotechnology, Hanseo University, Seosan 31962, Korea

**Keywords:** *Opuntia ficus-indica* var. *saboten*, glucose uptake, AMPK, p38 MAPK, GLUT4, *db/db* mice, L6 myoblasts

## Abstract

*Opuntia ficus-indica* var. *saboten* (OFS) has been used in traditional medicine for centuries to treat several illnesses, including diabetes. However, detailed mechanisms underlying hypoglycemic effects remain unclear. In this study, the mechanism underlying the hypoglycemic activity of OFS was evaluated using in vitro and in vivo systems. OFS treatment inhibited α-glucosidase activity and intestinal glucose absorption assessed by Na^+^-dependent glucose uptake using brush border membrane vesicles. AMP-activated protein kinase (AMPK) is widely recognized as an important regulator of glucose transport in skeletal muscle, and p38 mitogen-activated protein kinase (MAPK) has been proposed to be a component of AMPK-mediated signaling. In the present study, OFS dose-dependently increased glucose uptake in L6 muscle cells. The AMPK and p38 MAPK phosphorylations were stimulated by OFS, and inhibitors of AMPK (compound **C**) and p38 MAPK (SB203580) abolished the effects of OFS. Furthermore, OFS increased glucose transporter 4 (GLUT4) translocation to the plasma membrane. OFS administration (1 g/kg and 2 g/kg body weight) in *db/db* mice dose-dependently ameliorated hyperglycemia, hyperinsulinemia, and glucose tolerance. Insulin resistance assessed by homeostasis model assessment of insulin resistance and quantitative insulin sensitivity check index were also dose-dependently improved with OFS treatment. OFS administration improved pancreatic function through increased β-cell mass in *db/db* mice. These findings suggest that OFS acts by inhibiting glucose absorption from the intestine and enhancing glucose uptake from insulin-sensitive muscle cells through the AMPK/p38 MAPK signaling pathway.

## 1. Introduction

Type 2 diabetes mellitus (T2DM) has rapidly become the most prevalent chronic disease worldwide and remains one of the major health challenges of the 21st century. One of the therapeutic approaches for decreasing postprandial hyperglycemia is to retard the absorption of glucose by inhibition of carbohydrate-hydrolyzing enzymes such as α-amylase and α-glycosidase. However, they are not able to prevent glucose absorption when glucose itself has been ingested. Hence, it might be important to inhibit intestinal glucose absorption as well as inhibit glucosidase or amylase activity for the regulation of postprandial blood glucose level. Intestinal glucose absorption is thought to be regulated by the Na^+^-dependent glucose transporter 1 (SGLT1) at the apical membrane of the intestinal epithelia [1]. It has been shown in diabetic animals and humans that the capacity of the small intestine to absorb glucose increases at the brush border membrane vesicles (BBMVs) due to the enhanced activity and abundance of SGLT1 [2,3].

T2DM is characterized by insulin resistance of target tissues, caused by reduced transmission of insulin signaling, combined with progressive functional deterioration and increased death of insulin secreting pancreatic β-cells [4]. These two pathological processes are manifested by impaired glucose tolerance of affected individuals, leading to hyperglycemia, as well as other metabolic abnormalities. Once hyperglycemia becomes apparent, β-cell function progressively deteriorates: glucose-induced insulin secretion becomes further impaired and degranulation of β-cells becomes evident, often accompanied by a decrease in the number of β-cells [5].

Insulin is secreted in response to high blood sugar levels. It serves to stimulate glucose uptake and metabolism in peripheral tissues. Skeletal muscle is one of the important tissues for whole-body insulin-mediated glucose disposal in the postprandial state [6,7], and is, therefore, the most important site for whole-body glucose homeostasis. It is known that in subjects with T2DM, essentially all the impairment in insulin-mediated glucose disposal is caused by inadequate glucose uptake by muscle [8]. Thus, many researchers have investigated the effect of compounds that stimulate glucose uptake in skeletal muscle as a therapeutic target for metabolic disorders [9,10].

Glucose uptake is mainly mediated by glucose transporter 4 (GLUT4), a key determinant of whole-body glucose homeostasis, which is highly expressed in skeletal muscle and adipose tissue [11]. GLUT4 plays a pivotal role in acute glucose uptake in skeletal muscle, translocating from intracellular storage sites to the plasma membrane to incorporate glucose into the cells. The stimulation of GLUT4 translocation and glucose uptake is induced by the activation of adenosine monophosphate-activated protein kinase (AMPK) [12]. AMPK also increases the phosphorylation and activity of mitogen-activated protein kinase (MAPK) family components [13,14]; for example, p38 MAPK participates in the full activation of GLUT4 [15]. Activation of p38 MAPK participates in stimulation of glucose uptake by both insulin and contraction stimuli in skeletal muscles [16]. As AMPK activators, many natural and synthesized chemicals are also able to increase glucose uptake and improve hyperglycemia through activation of p38 MAPK [17,18].

For centuries a large number of botanical preparations have been used for blood glucose management. One promising traditionally used plant is the cactus *Opuntia* sp. Several species of the genus *Opuntia* (Cactaceae) grow extensively in desert or semi-desert regions in Mexico and the United States as well as in Mediterranean countries. Common names for the plant are “nopal” in Mexico, “prickly-pear cactus” in the Southern United States and “Indian fig cactus” in Europe. Its fruits and stems have been traditionally used in oriental folk medicines to treat diabetes, hypertension, asthma, burns, edema and indigestion [19,20]. The flattened, leaf-like stem, or cladode, is rich in pectin, mucilage, minerals, malic acid, vitamins, and antioxidants. The stems have been reported to exhibit a variety of pharmacological actions, including hypoglycemic, antioxidant, apoptotic, and anti-inflammatory activities [19,20,21,22,23,24].

*Opuntia ficus*-*indica* (OF), one of the traditionally used species, is widely cultivated in the southern part of Korea. Several clinical studies with the species *Opuntia streptacantha* have been performed supporting its use as an anti-hyperglycemic drug, whereas clinical data on OF are limited [23]. Based on recent reports, OF has been found to lower blood glucose and to increase basal plasma insulin levels in animals [25,26] as well as in humans [27,28]. However, the underlying mechanisms of the hypoglycemic activity have not been fully elucidated, and hence the molecular aspects of the mechanism behind the hypoglycemic and antidiabetic effects of OF need to be explored. With this background, the present study was carried out to exemplify the molecular mechanistic action of OF var. *saboten* (OFS) at the cellular level as well as in diabetic mouse strain of C57BL/6J-*db/db* (*db/db*) which exhibits many of the metabolic disturbances of human T2DM. The results of the present study revealed, for the first time, that OFS extract stimulated glucose transport in L6 myoblasts through GLUT4 translocation to the plasma membrane by activation of AMPK and its downstream target p38 MAPK.

## 2. Materials and Methods

### 2.1. Plant Materials

The stems of the *Opuntia ficus-indica* var. *saboten* (OFS) were purchased from Namhae Opuntia ficus indica Co. (Namhae, Korea), identified by one of the authors (Prof. KH Leem), and a voucher specimen was kept in the College of Korean Medicine, Semyung University (Voucher specimen No. SMH100414). OFS was washed, spines were manually removed, freeze-dried, and ground into powder. The dried powder was extracted two times with ten volumes of hot water (90 °C) for 10 h. The extracts were centrifuged, filtered, concentrated under vacuum, lyophilized, and subsequently used for the experiment.

### 2.2. α-Glucosidase Activity

The α-glucosidase activity was determined by a reaction in which α-glucosidase hydrolyzes *p*-nitrophenyl-α-d-glucopyranoside resulting in the formation of a colorimetric (405 nm) product, proportional to the α-glucosidase activity using commercially available kits (MAK123-1kt, Sigma-Aldrich, Saint Louis, MO, USA) based on yeast α-glucosidase. The α-glucosidase inhibitory activity was expressed as the inhibition percentage and was calculated as follows: inhibition (%) = (1 − ΔA_sam_/ΔA_con_) × 100%. Δ was defined as absorbance of the sample or the control. Acarbose (1 mg/mL) was used as a positive control.

### 2.3. Na^+^-Dependent Glucose Uptake in Brush-Border-Membrane Vesicle (BBMV)

BBMV were prepared using a previously described method with some modification [29]. All subsequent isolation steps were performed at 4 °C. Male ICR mice (9-week-old) jejunal mucosal scrapings were suspended in 10 mM HEPES/Tris buffer (pH 7.5) containing 300 mM mannitol and 300 mM MgCl_2_, and homogenized in a glass-Teflon homogenizer (Glass-Col, Terre Haute, IN, USA) for 10 min at 300 rpm. The mixture was centrifuged at 7500× *g* for 10 min at 4 °C. The supernatant was centrifuged at 27,000× *g* for 45 min. The resulting pellets were resuspended in 10 mM HEPES/Tris buffer containing 300 mM mannitol to a final protein concentration of 10 mg/mL, and stored in liquid nitrogen until use. The degree of purity in BBMV was assessed by the enrichment of alkaline phosphatase (ALP) in the finally prepared BBMV compared to the homogenate of intestinal scrapings. ALP activity was determined with ALP assay kit (Yeongdong Pharmaceutical Co., Seoul, Korea). The specific ALP activities of mucosal homogenate and BBMV suspension were 0.36 ± 0.03 and 2.34 ± 0.21 units/mg protein (mean ± SD), respectively, exhibiting 6.5-fold enrichment in final BBMV fraction. Protein was determined by using the Bio-Rad protein assay kit with bovine albumin as standard (Bio-Rad, Hercules, CA, USA).

Measurement of Na^+^-dependent glucose uptake by BBMV was determined by incubating 150 µL BBMV suspension with 850 µL uptake buffer containing 1 mM 2-(*N*-(7-nitrobenz-2-oxa-1,3-diazol-4-yl)amino)-2-deoxy-d-glucose (2-NBDG) and OFS (50–500 µg/mL) at 37 °C for 15 min. The uptake reaction was stopped by centrifuging for 20 min at 15,000 rpm, and the BBMV pellet was washed with stop buffer. The uptake and stop buffers were 10 mM HEPES/Tris (pH 7.5) containing 150 mM NaCl and 10 mM HEPES/Tris (pH 7.5) containing 150 mM KCl, respectively. Glucose uptake was measured by detecting fluorescence intensity of 2-NBDG.

### 2.4. Cell Culture

Rat L6 myoblasts were obtained from American Type Culture Collection (Rockville, MD, USA) and maintained at 37 °C under a humidified 5% CO_2_ atmosphere. Cells were grown in Dulbecco’s modified Eagle’s medium (DMEM) (Gibco BRL, NY, USA) supplemented with 10% fetal bovine serum (Gibco BRL) and 1% antibiotics (Gibco BRL). L6 myoblasts differentiation was induced by switching confluent cells to medium supplemented with 2% FBS. Experiments were performed in differentiated myoblasts.

### 2.5. Cell Viability

The cytotoxicity was assessed by the 3-(4,5-dimethyl-thiazol-2-yl)-2,5-diphenyl tetrazolium bromide (MTT, Sigma-Aldrich Inc., St. Louis, MO, USA) assay as described previously [30].

### 2.6. Glucose Uptake

Fully differentiated L6 myoblasts grown on 24-well plates (5 × 10^5^ cells/well) were incubated for 24 h with OFS. For inhibition of signal pathways, L6 myoblasts were pre-treated with 50 µM compound **C** (an AMPK inhibitor) or 5 µM SB203580 (p38 inhibitor) purchased from Sigma-Aldrich (New York, NY, USA) for 60 min. Glucose uptake was measured based on Yamada et al. [31]. Subsequently 100 µM of the fluorescent glucose analog 2-(*N*-(7-nitrobenz-2-oxa-1,3-diazol-4-yl)amino)-2-deoxyglucose (2-NBDG, Molecular Probes, NY, USA) was added for 15 min. After incubation, cells were washed with ice-cold PBS to remove free 2-NBDG and remaining fluorescence was measured with a fluorescence microplate reader (excitation 485 nm; emission 535 nm, FL×800, Bio-Teck, Portland, OR, USA). Insulin (100 nM) was used as a positive control.

### 2.7. Immunoblot Analysis

Differentiated L6 myoblasts treated with OFS were lysed, and equal amounts of protein were subjected to SDS-PAGE and immunoblotted with antibodies specific for AMPK, p-AMPK (Thr 172), ACC, p-ACC, JNK, p-JNK, ERK, p-ERK, p38, p-p38, GLUT4 and β-actin (Cell Signaling Technology, Beverly, MA, USA). Immunoreactive bands were visualized using chemiluminescent imaging system (Fusion SL2, Vilber Lourmat, Marne-la-Vallée Cedex, France), and analyzed by Bio1d software (Vilber Lourmat, Marne-la-Vallée Cedex, France).

### 2.8. Plasma Membrane Fractionation and GLUT4 Translocation Analysis

L6 cells were homogenized by sonication for 5 min at 40 kHz (UCP-20, JEIO Tech Co., Seoul, Korea) in ice-cold PBS and centrifuged at 700× *g* for 7 min to remove unhomogenized cellular debris and nuclei from the homogenate. The supernatant was harvested and centrifuged at 760× *g* for 10 min to remove mitochondria. Then, the supernatant was centrifuged at 35,000× *g* (ULTRA 5.0, Hanil Science Inc., Kimpo, Korea) for 60 min at 4 °C. The pellet was used as the plasma membrane fraction. The amounts of protein were quantified by using Protein Assay Kit (Bio-Rad Laboratories, Inc., Philadelphia, PA, USA) for Western blotting for GLUT4.

### 2.9. In Vivo Hypoglycemic Activity Evaluation in db/db Mice

Four-week-old male *db/db* (C57BL/6J *db/db*) and their non-diabetic heterozygous littermates (*db/-*) were purchased from Samtako (Osan, Korea). Mice were housed under temperature (22–26 °C) and light (12-h light/dark cycle) controlled conditions. After acclimation for 1 week, *db/db* mice were divided into 3 groups (*n* = 8); diabetic control (DC, vehicle), diabetic 1 g/kg OFS treated group (D1), and diabetic 2 g/kg OFS treated group (D2). Non-diabetic normal mice were divided into 2 groups (*n* = 8); normal control (NC, vehicle) and normal 1 g/kg OFS treated group (N1). OFS was dissolved in saline solution and was administered orally by gavage tube. The dosage of OFS was adjusted weekly based on the body weight change. The body weight and fasting blood glucose of each mouse were monitored weekly. All animal protocol used in this study was reviewed and approved by the Semyung University Institutional Animal Care and Use Committee (Smecae-16-05-02).

#### 2.9.1. Metabolic Parameters

Four weeks after feeding, oral glucose tolerance test (OGTT) was performed following overnight fast. OGTT was determined in response to oral administration of 2 g glucose/kg body weight. Blood glucose was measured from the tail vein 0, 15, 30, 60, and 90 min after glucose loading using one touch glucose measurement system (AGM-4000, Allmedicus, Anyang, Korea). Quantitative glucose tolerance of each group was calculated by the area under the curve (AUC) method using GraphPad Prism software version 6 (GraphPad Software Inc., La Jolla, CA, USA). Blood was collected and centrifuged (3000 rpm, 20 min, 4 °C). Serum insulin levels were measured using an ELISA kit (Thermo Fisher Scientific, Waltham, MA, USA). Homeostasis model assessment for insulin resistance (HOMA-IR) was calculated using the following formula: 

HOMA-IR (mM/L × µU/mL) = fasting glucose (mM/L) × fasting insulin (µU/mL)/22.5. Insulin sensitivity was calculated by quantitative insulin sensitivity check index (QUICKI) [32]: 1/[log fasting insulin (mU/L) + log fasting glucose (mg/dL)].

#### 2.9.2. Histological Examination

Pancreatic tissue fragments were fixed in 10% neutralized formalin solution for 24 h. After fixing in Bouin’s solution, the tissues were dehydrated, embedded in paraffin, and sectioned with 5 μm thickness (Leica, Wetzlar, Germany). Histological staining of β-cells using aldehyde fuchsin staining was performed [33].

### 2.10. Statistical Analysis

The data were expressed as mean ± SE. One-way ANOVA followed by Tukey’s post hoc test was performed for statistical analysis (GraphPad prism ver. 6), and p-values of less than 0.05 (*p* < 0.05) indicated significant differences. All in vitro experiments were performed with triplicate independent samples.

## 3. Results

### 3.1. Effects on α-Glucosidase Activity and Intestinal Glucose Uptake

**Figure 1:**
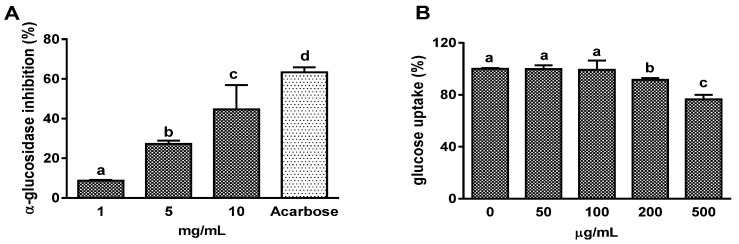
Effect of OFS (o*puntia ficus-indica* var*. saboten*) on the inhibition of α-glucosidase activity (**A**) and BBMV glucose uptake (**B**). Intestinal brush border membrane vesicles (BBMV) were isolated from jejunum of ICR mice, and Na^+^-dependent glucose uptake was determined. Glucose uptake data are expressed as the percentage of control group. Each value is the mean ± SE (*n* = 3). The bars with a different letter are significantly different from each other at the level of *p* < 0.05.

α-glucosidase inhibitory activity of OFS extract was detected by an enzyme activity assay in vitro. OFS inhibited enzyme activity in a concentration-dependent manner, and 10 mg/mL OFS inhibited 64.9% α-glucosidase activity reaching 78% of the acarbose effect (1 mg/mL, 83.5% inhibition) which is used as a positive control (Figure 1A). Next, the intestinal glucose uptake inhibitory activity of OFS extract was determined by in vitro model using 2-NBDG. Since intestinal glucose absorption is mediated predominantly by SGLT1 [1], the effect of OFS on intestinal Na^+^-dependent glucose uptake was determined in isolated jejunal BBMV, which has abundant expression of SGLT1. OFS extract at 200 and 500 µg/mL concentrations significantly suppressed Na^+^-dependent glucose uptake into the BBMV in a dose-dependent manner (Figure 1B). The inhibitory effects were 8.3% and 23.4% at 200 and 500 µg/mL, respectively.

### 3.2. Effect on Glucose Uptake and AMPK Activation in L6 Myoblasts

**Figure 2:**
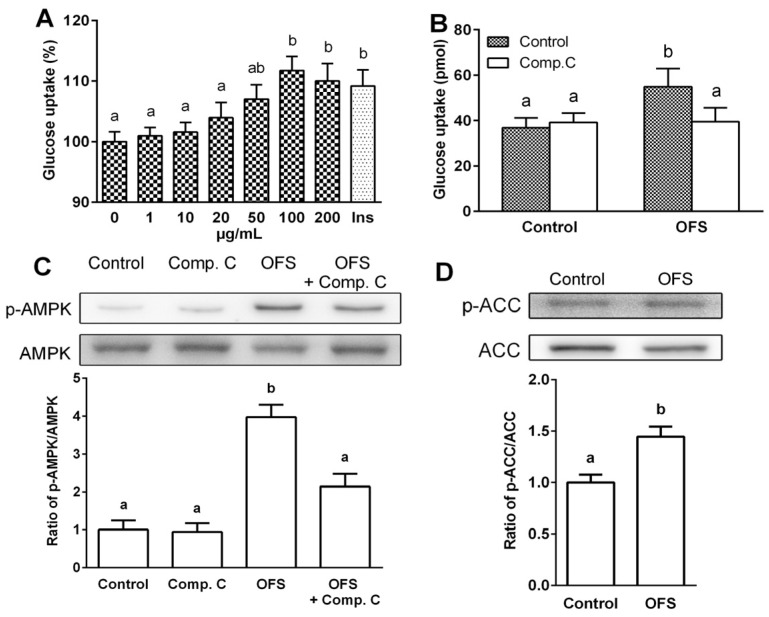
Effects of OFS on glucose uptake and AMP-activated protein kinase (AMPK) activation in L6 myoblasts. (**A**) Glucose uptake was determined in differentiated L6 cells using 2-NBDG; (**B**) Effect of AMPK inhibitor (compound **C**) on glucose uptake; (**C**,**D**) Effects of OFS on AMPK and acetyl-CoA carboxylase (ACC) phosphorylation. Cells were treated with OFS (100 µg/mL) and/or compound **C** (50 µM), and total cell lysates were used to determine the phosphorylation levels of AMPK and ACC by Western blotting. Each value is the mean ± SE (*n* = 3). The bars with a different letter are significantly different from each other at the level of *p* < 0.05.

Skeletal muscle, a key insulin sensitive tissue is the main site of postprandial glucose utilization and a major element in the maintenance of glucose homeostasis. Thus, augmenting glucose uptake by skeletal muscle may attenuate insulin resistance, which precedes type 2 diabetes. To determine whether OFS extract improves glucose uptake in muscle cells, 2-NBDG uptake by differentiated L6 myoblasts was measured in the presence or absence of OFS at various concentrations for 1 h. As shown in Figure 2A, OFS extract enhanced glucose uptake in a dose-dependent manner reaching peak value at 100 µg/mL. OFS induced an 11.7% increase in glucose uptake in L6 myoblasts compared with control while a 9.1% increase was observed with insulin. OFS extract did not influence the viability of L6 cells in dose ranges between 0 and 200 µg/mL, as assessed by MTT assay (data not shown). There was also no indication of cellular toxicity with respect to changes in cell morphology or appearance under the phase-contrast microscope.

It is known that activation of AMPK plays an important role in increasing glucose uptake [12]. To clarify the role of AMPK on OFS-induced glucose uptake, compound **C**, an ATP-competitive inhibitor of AMPK was used. Pretreatment of cells with compound **C** in the presence of OFS completely blocked OFS-mediated glucose uptake (Figure 2B). Next, the activation of AMPK and its downstream molecule, acetyl-CoA carboxylase (ACC) were examined using immunoblotting analysis. In differentiated L6 myoblasts, OFS treatment stimulated the phosphorylation of AMPK compared with control, and compound **C** inhibited the OFS-mediated phosphorylation of AMPK (Figure 2C). The levels of phospho-ACC were also higher in OFS-treated cells than control cells. This result suggested that the stimulatory effect of OFS on glucose uptake is, at least partly, mediated by the AMPK pathway.

### 3.3. Effect on MAPK Activation in L6 Myoblasts

**Figure 3:**
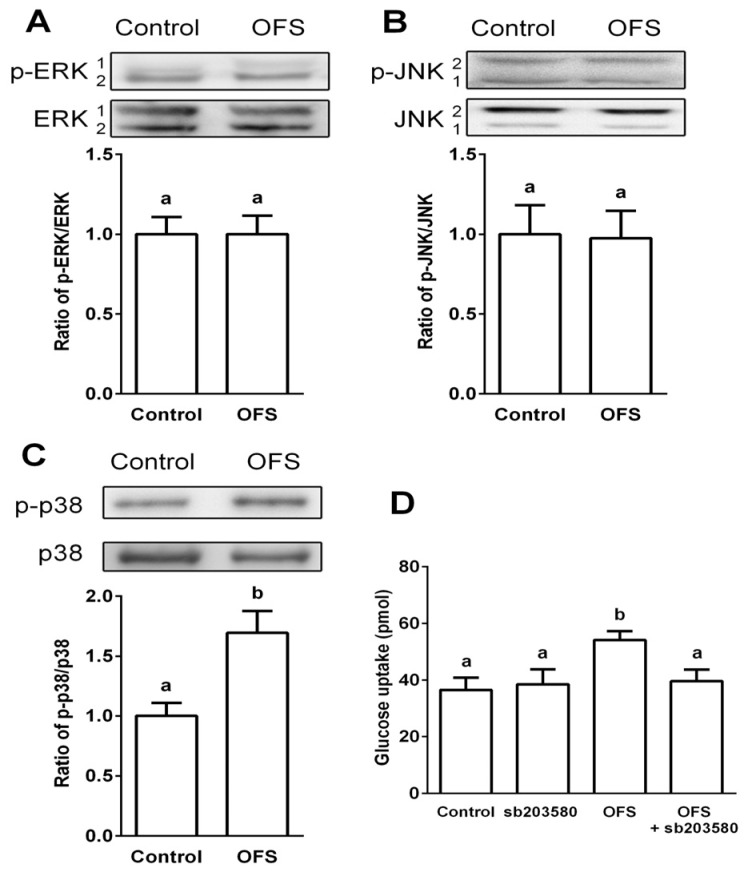
Effects of OFS on activation of MAPKs in L6 myoblasts. Western blot and densitometric results of the phosphorylation of ERK1/2 (**A**), JNK1/2 (**B**), and p38 (**C**). Effect of p38 inhibitor (sb203580) on glucose uptake (**D**). Cells were treated with OFS (100 µg/mL), and total cell lysates were used to determine the phosphorylation levels of MAPKs by Western blotting. Each value is the mean ± SE (*n* = 3). The bars with a different letter are significantly different from each other at the level of *p* < 0.05.

It has been reported that insulin activation leads to the phosphorylation and activation of members of the MAPK family, specifically ERK1/2, JNK and p38 MAPK, which phosphorylate and control the activity of other downstream protein kinases and transcription factors [34]. Also, p38 MAPK has been reported as a downstream target of AMPK and a key target involved in glucose uptake [14]. To verify the role of MAPKs in OFS-mediated glucose uptake, the effect of OFS on the activation of three different MAPKs (ERK1/2, JNK1/2, and p38) were examined. Immunodetection of phosphorylated MAPKs were used to determine the levels of activated MAPKs. OFS treatment (100 µg/mL) failed to affect the phosphorylation of ERK1/2 and JNK1/2 while p38 MAPK phosphorylation was significantly increased (49% increase) by OFS treatment. Furthermore, pretreatment of cells with SB203580, an inhibitor of p38, completely blocked OFS-mediated glucose uptake (Figure 3D).

### 3.4. Effect on GLUT4 Translocation to the Plasma Membrane in L6 Myoblasts

**Figure 4:**
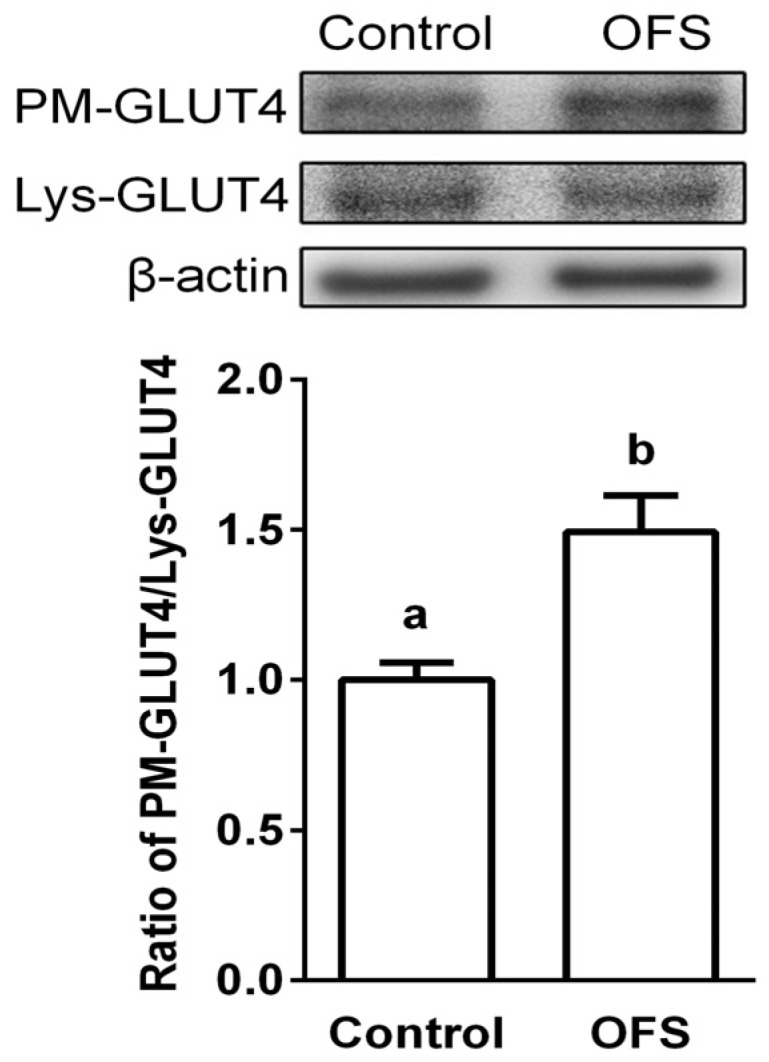
Effects of OFS on GLUT4 translocation to the plasma membrane in L6 myoblasts. Cells were treated with OFS, and total cell lysates (Lys) and plasma membrane fraction (PM) were analyzed by west blotting. Each value is the mean ± SE (*n* = 3). The bars with a different letter are significantly different from each other at the level of *p* < 0.05.

It is known that AMPK and p38 activation causes GLUT4 translocation to plasma membrane, and glucose uptake in skeletal muscle is accompanied by an increase in GLUT4 translocation [12]. Thus, the molecular mechanism of OFS-induced glucose uptake was investigated by the localization of GLUT4 in the plasma membrane using Western blotting analyses. OFS stimulated the translocation of GLUT4 to the plasma membrane without alteration of the expression level of GLUT4 (Figure 4) in cell lysates, suggesting that GLUT4 translocation is responsible for OFS-induced glucose uptake. The ratio of plasma membrane fraction GLUT4 to total lysates GLUT4 was increased by 49% in plasma fractions of OFS treated L6 myoblasts. These results suggest that increased glucose uptake induced by OFS might be mediated through activation of both AMPK and p38 MAPK, and the subsequent increase in GLUT4 translocation in skeletal muscles.

### 3.5. Preventive Effect on Progression of Diabetes in db/db Mice

**Figure 5:**
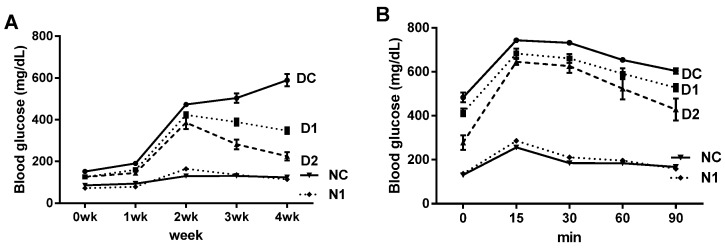
Effect of OFS on blood glucose levels during experimental period (**A**) and oral glucose tolerance (**B**) in *db/db* mice. Normal *db/-* mice control (NC); normal *db/-* mice fed 1 g/kg body weight of OFS (N1); *db/db* mice control (DC); *db/db* mice fed 1 g/kg body weight of OFS (D1); *db/db* mice fed 2 g/kg body weight of OFS (D2). For glucose tolerance tests, mice were given glucose (2 g/kg body weight). Data are expressed as means ± SE (*n* = 8). Bars not sharing a common letter are significantly different at *p* < 0.05.

The preventive effects on the progressive hyperglycemia of diabetic mice were examined in *db/db* mice and their non-diabetic littermates. Body weight and food intake were significantly higher in DC group compared with normal mice, and administration of OFS had no significant effect on body weight and food intake in diabetic mice (data not shown). As shown in Figure 5A, *db/db* mice showed a continuous increase in blood glucose throughout the experiment. However, OFS treated group tended to show lower glucose levels during the experiment. In the final week of the experiment, blood glucose levels were significantly decreased in OFS treated *db/db* mice in a dose-dependent manner resulting in 62% decrease in D2 group (2 g/kg body weight OFS treated *db/db* mice) compared with DC group (Figure 5A and Table 1). Oral glucose tolerance test (OGTT), which can be used to evaluate blood glucose homeostasis, was performed. As seen in Figure 5B, glucose load in non-diabetic mice produced rapid increase in blood glucose levels reaching peak value at 15 min and returned to baseline values within 90 min. In contrast, DC mice demonstrated basal hyperglycemia which remained significantly higher level during all time points determined compared with normal control (NC) mice. While DC mice were glucose intolerant, OFS administered mice showed significantly and dose-dependently lower blood glucose levels at fasted baseline and at every time point thereafter exhibiting definite improvement in glucose homeostasis. The areas under the curve (AUC) of blood glucose were markedly lower in the non-diabetic mice than the diabetic mice, and OFS treatment dose-dependently decreased AUC value in diabetic mice (Table 1).

Plasma insulin levels of diabetic mice were more than 2-fold higher than non-diabetic mice indicating insulin resistance in type 2 diabetes (Table 1). OFS administration reduced insulin levels in diabetic mice, although dose-dependency was not observed. Homeostatic model assessment of insulin resistance (HOMA-IR), an indicator of insulin resistance, was also significantly improved by OFS treatment in a dose-dependent manner. QUICKI, an insulin sensitivity index suggested by Katz et al. [32], was significantly higher in normal mice than diabetic mice, and OFS treatment dose-dependently improved insulin sensitivity in *db/db* mice. During the experimental period, OFS treatment did not induce a difference in the body weight, blood glucose and plasma insulin levels in normal mice, suggesting that OFS had no adverse side effects on normal mice. Taken together, these results clearly indicated an improvement in insulin resistance and insulin sensitivity in *db/db* mice treated with OFS.

### 3.6. Effect on Regeneration of β-Cells in db/db Mice

**Figure 6:**
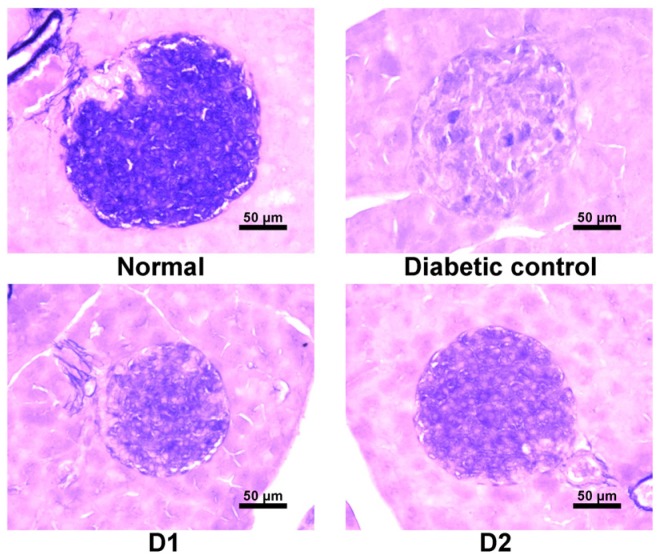
Photomicrograph of pancreatic islet of normal, diabetic, and OFS treated mice stained with aldehyde fuchsin. Normal *db/-* mice control (NC); *db/db* mice control (DC); *db/db* mice fed 1 g/kg body weight of OFS (D1); *db/db* mice fed 2 g/kg body weight of OFS (D2). β-cells in pancreatic islets stained purple-violet. Bar = 50 µm.

Since OFS treatment exhibited antidiabetic effect on *db/db* mice, histological changes in the pancreas was further examined. A correlation has been reported between the aldehyde fuchsin staining and the number of pancreatic β-granules, and the intensity of this staining has been used as a histochemical index of the content of insulin in the β-cells [33]. Figure 6 shows photomicrographs of aldehyde fuchsin histochemical staining of pancreas in OFS treated diabetic mice. Significant histological differences were noted between *db/db* mice and normal littermates. Pancreas in normal mice possessed normal islets with clusters of purple granulated β-cells, while atrophy of the islets as well as degenerative changes of β-cells were observed in *db/db* mice compared with normal mice. In contrast, treatment with OFS dose-dependently protected marked cellular degeneration of β-cells and exhibited significant increase in cellularity of islet and regeneration. Compared with the *db/db* mice, the morphology of the islet became regular, cells in the islet were relatively orderly arranged, and staining in β-cell was darker in the *db/db* mice receiving OFS.

## 4. Discussion

There have been a number of in vivo studies showing that *Opuntia ficus*-*indica* improves diabetic condition by reducing blood glucose and increasing blood insulin level [24,25,26,27,28]. However, most of these studies used mixture of stem, fruit, and/or medicinal herbs, and the detailed mechanism of the hypoglycemic activity has not yet been fully elucidated. In the present study, the underlying mechanism of the hypoglycemic effect of *Opuntia ficus*-*indica* var. *saboten* (OFS), one of the *Opuntia ficus-indica* species widely cultivated in the southern part of Korea was examined. Since the primary symptom of T2DM is elevated blood glucose due to either lack of insulin or insulin resistance, the present study focused on the factors which modulate blood glucose levels, including postprandial glucose control and glucose uptake in L6 myoblasts. To investigate the effect of OFS on T2DM in vivo, insulin resistance/sensitivity, assessed by oral glucose tolerance, were determined and pancreatic β-cell function in *db/db* mice were further examined. In addition, the detailed molecular basis underlying the hypoglycemic effect of OFS by focusing on the AMPK/MAPK signaling pathways mediating glucose uptake as well as GLUT4 translocation in L6 myoblasts was examined. The principal finding of this study is that a hot-water extract of OFS had a metabolic effect on skeletal muscles and improved glucose resistance/sensitivity. Specifically, the present study demonstrated for the first time that the AMPK/p38 MAPK signaling pathway and GLUT4 translocation to the plasma membrane were involved in OFS-stimulated glucose metabolism in muscle cells.

The importance of postprandial glucose control in the development of diabetic complications is widely recognized and several inhibitors of α-amylase or β-glucosidase were proposed to control postprandial hyperglycemia. However, the inhibitors of these enzymes are not able to prevent glucose absorption when glucose itself has been ingested. Therefore, inhibition of intestinal glucose absorption as well as glucosidase and/or amylase activity is important for the regulation of postprandial blood glucose level. In the present study, an in vitro study was conducted to examine the effects of OFS on α-glucosidase inhibitory activity and intestinal BBMV glucose uptake. The results revealed that OFS dose-dependently inhibited α-glucosidase activity and reduced intestinal Na^+^-dependent glucose uptake in BBMV.

The main function of insulin is to control glucose homeostasis by stimulating glucose uptake in peripheral tissues, especially in insulin targeted skeletal muscle, and insulin resistance in T2DM is manifested by decreased insulin-stimulated glucose uptake and impaired metabolism in adipocytes and skeletal muscle [35]. It is known that activation of AMPK and p38 MAPK can increase glucose uptake through increasing GLUT4 translocation to cell membranes. Activation of AMPK induces the recruitment of GLUT4 to the plasma membrane resulting in up-regulated glucose uptake [12,14], and several studies have demonstrated that AMPK and its signaling pathway are potential molecular targets in the development of drugs for the treatment of type 2 diabetes and obesity [36,37]. Previous studies also indicated that p38 MAPK is a downstream component of the AMPK signaling pathway [12,14,15], and is essential for maximal increases in glucose uptake by insulin treatment and contractions of skeletal muscle cells [16]. The present study demonstrated that OFS extract dose-dependently increased glucose-uptake in L6 myoblasts (Figure 2) and stimulated the translocation of GLUT4 to the plasma membrane (Figure 6). To clarify the underlying mechanism of the OFS-mediated glucose metabolism, AMPK and p38 inhibitor, compound **C** and SB203580, respectively, was used. AMPK inhibitor potently reduced the OFS-stimulated glucose uptake and OFS-induced AMPK activation (Figure 2B,C). p38 MAPK inhibitor, SB203580, completely blocked the OFS-induced glucose uptake (Figure 3D). These data clearly suggest that AMPK and p38 MAPK are involved in the stimulation of glucose uptake by OFS. Taken together, these in vitro results suggest that OFS acts at intestinal level by delaying and/or inhibiting glucose absorption and at a peripheral level on insulin-sensitive tissues by facilitating the entry of glucose into muscle cell through AMPK/p38 MAPK signalling pathway.

The hypoglycemic effect was further evaluated in vivo using genetically diabetic *db/db* mice. In an insulin resistant state, as in *db/db* mice, insulin does not promote glucose uptake and utilization effectively, so insulin secretion increases excessively with compensation to maintain blood glucose homeostasis [38]. Treatment with OFS significantly and dose-dependently ameliorated hyperglycemia and improved glucose handling capacity. Moreover, the HOMA-IR index, a parameter for evaluating the degree of insulin resistance, and quantitative insulin sensitivity check index (QUICKI) were significantly reversed in a dose-dependent manner by the administration of OFS in *db/db* mice (Table 1). A further indication of improved peripheral glucose disposal and insulin sensitivity in the OFS fed *db*/*db* mice was provided by the improved glycemic response to OGTT (Figure 5). Skeletal muscle insulin resistance is a common defect in T2DM because nearly 90% of the insulin mediated glucose is taken up by skeletal muscle [38]. In the present study, increased glucose uptake in L6 myoblasts together with the significantly improved OGTT, supported the hypothesis that OFS exerts direct effects at sites of peripheral insulin action.

Diabetic *db/db* mice are characterized by hyperglycemia, lower pancreatic insulin stores, and higher plasma insulin levels resulting in pancreatic β-cell necrosis and atrophy [39]. The histochemical examination revealed the protective effects of OFS in pancreatic islet function (Figure 6). Compared with diabetic mice, normalized β-cell regeneration as well as functional integrity islet morphology was observed in OFS treated mice. Prolonged exposure of pancreatic β-cells to high glucose levels is known to cause β-cell dysfunction, called glucose toxicity [40], and these damaged β-cells often display extensive degranulation, and are clinically associated with the development of diabetes in some model animals for T2DM [39] including *db/db* mice. Therefore, the ameliorative effect of OFS on hyperglycemia seen in *db/db* mice may contribute to restoration of β-cell function. The effect of OFS may be explained by the fact that OFS may reduce demands for excessive insulin secretion, preventing pancreatic exhaustion. On the other hand, normalization of glucose level by OFS may reduce functional demand of serum insulin levels.

The active compounds responsible for hypoglycemic activity of OFS have not yet been clarified. However, the diverse activities may be due to several different chemical compounds found in the extract which act in concert to bring about the hypoglycemic effects. The stems of OFS are rich in pectin, mucilage, and antioxidants such as polyphenols. Therefore, the hypoglycemic effects can be due to decreased intestinal glucose absorption provoked by high dietary fiber content of OFS. Dietary fiber content of OFS used in this study was about 56% (dry weight basis) similar to the report of Bensadón et al. [41] showing 60%–64% OF. Also, the water holding capacity of the gel-like mucilage of OFS might play an additional role. Soluble dietary fiber has been shown to exert hypoglycemic effects mediated through inhibition of carbohydrate digestion and absorption, and enhancement of peripheral insulin action [42]. In addition, antioxidants such as *N*-acetylcysteine, vitamin C and α-lipoic acid have been reported to be effective in reducing diabetic complications [43]. Fresh stems of Mexican nopal, one of the OFS species, have been reported to contain 7–22 mg ascorbic acid and 11.3–53.5 µg total carotenoids [44]. Antioxidative flavonoids, quercetin and its derivatives were isolated from the stems of OFS [45]. Avila-Nava et al. [46] reported the ROS scavenging activity of stem extract, and isolated several polyphenols such as quercetin, isorhamnetin and kaempferol. Several antioxidants have been reported to activate AMPK. Dietary vitamin E and C supplementation attenuated glucose intolerance in rats by AMPK activation in muscle [47]. Polyphenols such as resveratrol have been reported to activate the AMPK signaling pathway [48]. Therefore, water soluble dietary fiber as well as antioxidants such as vitamin c and polyphenols/flavonoids, exist in OFS extract and might play role in intestinal glucose absorption, glucose uptake to peripheral tissues, and improvement of insulin action in this study.

## 5. Conclusions

**Figure 7:**
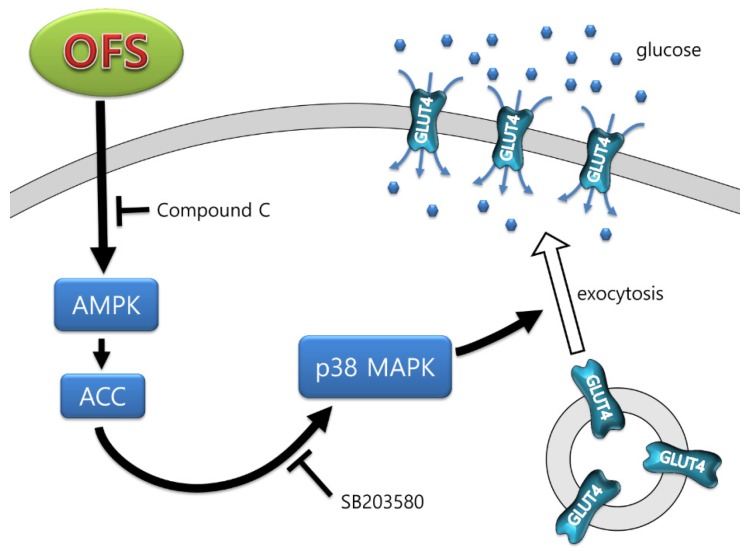
Schematic summary of the possible molecular mechanism by which OFS regulates glucose uptake in muscle cell.

In conclusion, the present study demonstrated that OFS improves glucose resistance/sensitivity and glucose uptake through a mechanism that involves AMPK/p38 MAPK signaling pathway and GLUT4 translocation from intracellular storage vesicles to the plasma membrane in muscle cells (Figure 7). Further study is necessary to identify the active compounds responsible for hypoglycemic activity of OFS, and better understand their mode of action.

## Figures and Tables

**Table 1:** Effects of OFS on glycemic index parameters.

Groups	DC	D1	D2	NC	N1
Fasting glucose (mg/dL)	589.3 ± 29.7 ^a^	347.8 ± 15.4 ^b^	224.8 ± 19.7 ^c^	124.6 ± 5.8 ^d^	114.6 ± 2.7 ^d^
Insulin (µU/mL)	36.8 ± 4.1 ^a^	26.3 ± 2.2 ^b^	23.6 ± 2.6 ^b^	16.9 ± 1.9 ^c^	17.5 ± 4.7 ^c^
AUC ^1^	3218.3 ± 337.0	2841.3 ± 54.3	1451.7 ± 72.5	965.0 ± 6.3	2022.5 ± 10.6
HOMA-IR ^2^	43.95 ± 3.2 ^a^	25.25 ± 2.1 ^b^	16.43 ± 1.4 ^c^	5.47 ± 0.3 ^d^	5.88 ± 0.4 ^d^
QUICKI ^3^	0.24 ± 0.005 ^a^	0.25 ± 0.004 ^a^	0.26 ± 0.004 ^b^	0.30 ± 0.003 ^c^	0.29 ± 0.004 ^d^

Values are means ± SE (*n* = 8). Means in same row with different superscripts are significantly different (*p* < 0.05). ^1^ Area under the glucose tolerance test (arbitrary unit). ^2^ Homeostatic model assessment of insulin resistance. ^3^ Quantitative insulin sensitivity check index.
